# Delays in HIV-1 infant polymerase chain reaction testing may leave children without confirmed diagnoses in the Western Cape province, South Africa

**DOI:** 10.4102/ajlm.v11i1.1485

**Published:** 2022-06-23

**Authors:** Kamela L. Mahlakwane, Wolfgang Preiser, Nokwazi Nkosi, Nasheen Naidoo, Gert van Zyl

**Affiliations:** 1 Division of Medical Virology, Faculty of Medicine and Health Sciences, Stellenbosch University, Cape Town, South Africa; 2 Division of Medical Virology, Tygerberg Hospital, National Health Laboratory Service, Cape Town, South Africa; 3 Division of Clinical Pathology, Faculty of Medicine and Health Sciences, Stellenbosch University, Cape Town, South Africa; 4 Division of Clinical Pathology, Tygerberg Hospital, National Health Laboratory Service, Cape Town, South Africa

**Keywords:** infant HIV PCR, confirmatory testing, early infant diagnosis, EID, laboratory diagnosis, antiretroviral therapy, turn-around time, follow-up testing

## Abstract

**Background:**

Early diagnosis and confirmation of HIV infection in newborns is crucial for expedited initiation of antiretroviral therapy. Confirmatory testing must be done for all children with a reactive HIV PCR result. There is no comprehensive data on confirmatory testing and HIV PCR test request rejections at National Health Laboratory Service laboratories in South Africa.

**Objective:**

This study assessed the metrics of routine infant HIV PCR testing at the Tygerberg Hospital Virology Laboratory, Cape Town, Western Cape, South Africa, including the proportion of rejected test requests, turn-around time (TAT), and rate of confirmatory testing.

**Methods:**

We retrospectively reviewed laboratory-based data on all HIV PCR tests performed on children ≤ 24 months old (*n* = 43 346) and data on rejected HIV PCR requests (*n* = 1479) at the Tygerberg virology laboratory over two years (2017–2019). Data from sample collection to release of results were analysed to assess the TAT and follow-up patterns.

**Results:**

The proportion of rejected HIV PCR requests was 3.3%; 83.9% of these were rejected for various pre-analytical reasons. Most of the test results (89.2%) met the required 96-h TAT. Of the reactive initial test results, 53.5% had a follow-up sample tested, of which 93.1% were positive. Of the initial indeterminate results, 74.7% were negative on follow-up testing.

**Conclusion:**

A high proportion of HIV PCR requests were rejected for pre-analytical reasons. The high number of initial reactive tests without evidence of follow-up suggests that a shorter TAT is required to allow confirmatory testing before children are discharged.

## Introduction

The early diagnosis of HIV infection in infants is very important.^1,2^ Infants who are initiated on antiretroviral therapy (ART) within seven days of life are four times more likely to achieve early viral suppression than those who commence ART later.^3,4^ The South African national guidelines on the prevention of mother-to-child transmission of HIV recommend that HIV polymerase chain reaction (PCR) tests be conducted within 3–6 days of birth and that ART should be immediately initiated in all children with detectable HIV nucleic acid while awaiting the follow-up HIV PCR results.^5^

Early initiation of ART is associated with improved virological, immunological, and clinical outcomes.^6,7,8,9,10^ Early diagnosis of HIV in infants is done by testing for viral nucleic acid (DNA and/or RNA) in blood samples, usually by PCR.^11^

Current guidelines, including those issued by the Western Cape Department of Health and South African National Department of Health, recommend the testing of all HIV-exposed infants by PCR within seven days of birth (i.e., birth PCR), at approximately 10 weeks of age for those who tested negative at birth, at 18 weeks for high-risk infants who received extended prophylactic ART for 12 weeks, at six months, and after cessation of breastfeeding.^5,12,13^ The post-breastfeeding HIV test may either be by PCR if the child is younger than 18 months of age or by serology if the child is older.^5,11,12^

The World Health Organization recommends that HIV antibody testing be done after at least three months post-breastfeeding to allow for HIV antibody development and to prevent missing HIV infection in the last few days of breastfeeding.^13^ A negative antibody test in an infant older than 18 months is confirmation that the infant is not infected.^13^

Although the World Health Organization recommends HIV PCR testing for infants ≤ 18 months old, the age cut-off for HIV PCR testing at National Health Laboratory Service (NHLS) laboratories is 24 months rather than 18 months. This is based on studies that showed that maternal HIV antibody clearance took longer than 18 months in some perinatally exposed infants, with seroreversion rates of 89.3%, 94.2%, and 100.0% at ages 12, 18, and 24 months, respectively.^14,15^ Nevertheless, this policy has not been formalised in the laboratory diagnostic booklet, the Western Cape provincial testing guidelines, or the national Department of Health HIV testing guidelines.

Given the consequences of HIV infection (such as lifelong ART), a follow-up sample must be collected as soon as possible from all children with a reactive infant PCR result to confirm the diagnosis or detect false-positive initial results.^11,16^ However, a negative test result following an initial reactive result does not necessarily constitute false positivity, particularly in infants who are on prophylaxis or ART.

Clinical actions taken based on false-positive results may have dire biomedical (exposure to drug toxicity and side effects), psychosocial (possible stigma and negative impact on life outlook and future relationships), financial (ART costs), and medico-legal implications.^16^ Once initiated on ART, it may become impossible to distinguish a virally suppressed child from one who was never infected.^4^

In South Africa, the NHLS provides laboratory testing for the public health sector, providing healthcare for approximately 85% of the population who do not have medical insurance.^17^ The NHLS aims to issue results for at least 80% of infant PCR tests within a 96-h turn-around time (TAT).^18^ This TAT is defined by the NHLS^19^ as the time interval between sample reception and the release of results by a virologist (i.e., laboratory TAT). This TAT does not consider the periods between sample collection and reception, or between the review of results and the receipt of results by the patient. For this study, we refer to the period between sample collection and authorisation of results as ‘clinical TAT’.

The NHLS performs all HIV early infant diagnosis testing using the Roche^®^ COBAS^®^ AmpliPrep/COBAS^®^ TaqMan^®^ system (Roche^®^ Molecular Systems, Inc., Branchburg, New Jersey, United States) in a network of centralised laboratories.^17^ Results of this assay could be positive, negative, or indeterminate. Indeterminate qualitative HIV PCR results on the Roche^®^ COBAS^®^ AmpliPrep/COBAS^®^ TaqMan^®^ system were first described in the Tygerberg Hospital Virology Laboratory in 2012.^20,21^ During the period covered by this study, indeterminate results were defined as a cycle threshold of ≥ 33, or a relative fluorescence intensity below five.^22^ These diagnostic criteria have since been revised,^23,24,25,26^ mostly based on collated data, which showed cycle threshold value cut-offs to be the best predictor of reproducible positive results. The cycle threshold value of 33 was shown to most precisely differentiate clear positive from irreproducible cases.^23^

In the Tygerberg virology laboratory, HIV PCR testing is done only once on each sample unless the results are invalid, in which case a single retest is done on the remnant sample. Samples that produce invalid results for the second time are rejected with this reason attached: ‘failed after repeated attempts’. All other results (i.e., negative, positive, or indeterminate) are released as such, with no repeat testing of the remnant sample.

The World Health Organization reports that most women and their newborn infants are likely to be discharged within one to two days following uncomplicated vaginal delivery, and within two to four days following uncomplicated caesarean delivery.^27^ Thus, infant birth HIV PCR results should, ideally, be available before the child gets discharged to allow immediate optimal management (prophylaxis, ART, or confirmatory testing). HIV PCR TAT strategies should thus seek to address this challenge.

In this study, a laboratory-based retrospective review was conducted to determine the proportion of rejected infant HIV PCR requests, assess laboratory conformance with TAT requirements, and assess whether there were confirmatory tests done following reactive infant HIV PCR results performed on the Roche^®^ COBAS^®^ AmpliPrep/COBAS^®^ TaqMan^®^ system.

## Methods

### Ethical considerations

This study was approved by the Health Research Ethics Committee (HREC) of Stellenbosch University (reference number: S19/03/053). The HREC does not require patient consent when residual clinical samples are used for research if the research findings will not impact the patient or change their management in any way. Access to the extracted laboratory data was limited to the researchers.

### Study design and setting

This is a retrospective review of data generated at the Tygerberg Hospital Virology Laboratory in Cape Town, Western Cape, South Africa, between July 2017 and June 2019. The data was downloaded from the laboratory information system (LIS) of the Tygerberg Laboratory (TrakCare Lab, Intersystems Corporation, Cambridge, Massachusetts, United States).

The Tygerberg virology laboratory renders diagnostic virological pathology services to Tygerberg Hospital and other health facilities from its drainage areas. All samples received in the laboratory go through the routine process of sample reception, registration and analysis, as well as review of results.

All HIV-1 qualitative PCR results from ethylenediaminetetraacetic acid-anticoagulated whole blood and dried blood spot samples from children aged ≤ 24 months that were tested at the Tygerberg virology laboratory between 01 July 2017 and 30 June 2019 were included in this study. Samples from patients older than 24 months (*n* = 534) or with unknown age (*n* = 287), quality control samples (*n* = 29), and samples with coded names or surnames (*n* = 15) were excluded. A total of 43 346 tests were included in this study.

### Data collection

#### Rejection of test requests

A raw data set of rejected HIV PCR requests for children aged ≤ 24 months was extracted from the LIS (*n* = 1479) and assessed to determine the pattern of HIV PCR request rejections by the laboratory. Infant PCR test requests may be rejected for various reasons, as included in the LIS and NHLS standard operating procedure for sample rejection. These reasons may be pre-analytical (e.g. ‘unsuitable age’ for children aged > 24 months) or analytical (e.g. ‘laboratory error’).

Samples from other peripheral laboratories are referred both digitally and physically. Digital referrals that are not accompanied by the sample are rejected as ‘lost in transit’ or ‘specimen not received’ after consultation with the referring laboratory or the requesting clinician. All ethylenediaminetetraacetic acid-anticoagulated samples are discarded after seven days from the date of collection. Samples that are not tested within this period are rejected as ‘sample too old’. The term ‘insufficient specimen’ refers to remnant samples with initial invalid results and insufficient sample volume for repeat testing. The classification of these rejections as pre-analytical is, therefore, arbitrary.

#### Turn-around time

Laboratory TAT was calculated based on the NHLS definition,^19^ while clinical TAT was analysed as earlier defined. The time points were extracted from the LIS as recorded during each step of the testing process. The results were then stratified using a 96-h TAT cut-off.

#### Follow-up testing

All results were grouped using the unique laboratory code, that is, the medical record number, which is assigned to each patient and remains the same for all subsequent test requests. The time-to-follow-up was calculated as the number of days between the first and second sample collection dates as recorded on the LIS. In line with the guidelines, all positive and indeterminate results were expected to have a follow-up sample sent for confirmatory testing as soon as possible, while all the negative samples were expected to be followed up as per schedule. Negative follow-up test results were further checked to determine if a third follow-up sample was tested.

### Data analysis

Data were analysed using Microsoft Excel 2010 version 14 (Microsoft Corporation, Redmond, Washington, United States). Rejected test requests were stratified by reason for rejection and further summarised into pre-analytical and analytical reasons for rejection.

## Results

### Rejected test sets

**FIGURE 1:**
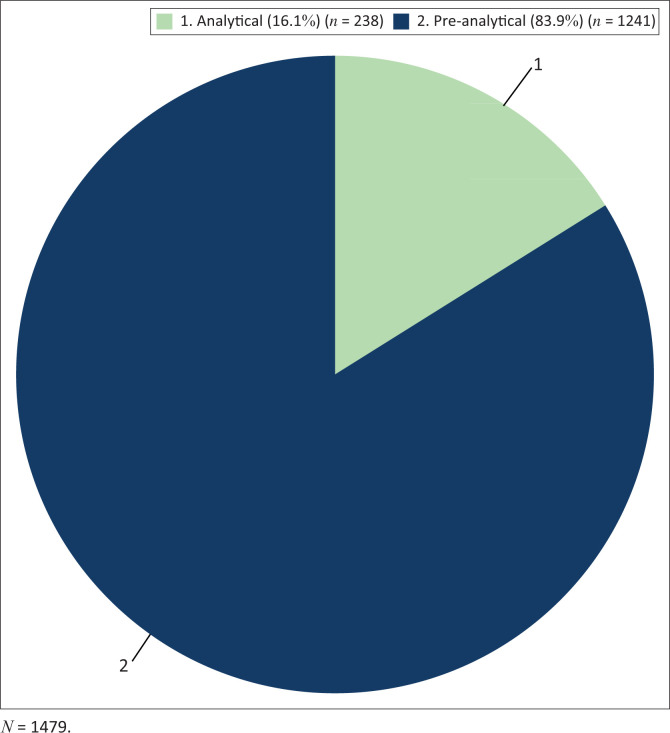
Pre-analytical and analytical reasons for HIV polymerase chain reaction test rejection at Tygerberg Hospital, Cape Town, South Africa, between July 2017 and June 2019.

Out of the 1479 rejected samples, 1241 (83.9%) were rejected for pre-analytical reasons, and 238 (16.1%) for analytical reasons (Figure 1). ‘Duplicate requests’ (21.3%), ‘insufficient specimen’ (21.1%), and ‘specimen not received’ (16.1%) were the most common pre-analytical reasons for sample rejections (Table 1). Other reasons that each constituted less than 1% of the reasons for test rejection were grouped as ‘other various reasons’.

**TABLE 1:** Reasons for rejection of HIV polymerase chain reaction test requests at Tygerberg Hospital, Cape Town, South Africa, between July 2017 and June 2019.

Reasons for rejection	No. of rejected requests	Percentages	Category
Duplicate request	315	21.3	Pre-analytical
Insufficient specimen	312	21.1	Pre-analytical
Specimen not received	238	16.1	Pre-analytical
Require blood specimen	106	7.2	Pre-analytical
Sample too old	50	3.4	Pre-analytical
Specimen not labelled	50	3.4	Pre-analytical
Information mismatch	46	3.1	Pre-analytical
Container empty	43	2.9	Pre-analytical
Invalid result	110	7.4	Analytical
Failed after repeated attempts	109	7.4	Analytical
Other various reasons†	100	6.8	Mixture

**Total rejected samples**	**1479**	**100.0**	**-**

† , Other reasons that each constituted ≤ 1% of the rejection reasons were grouped as ‘Other various reasons’. These rejection reasons include both pre-analytical and analytical reasons.

### Demographics

Of the 43 346 samples that were tested for infant HIV PCR, 27 978 (64.5%) were the patients’ first or initial HIV PCR samples, while 18 393 (42.4%) samples were collected at birth. Among the 9585 patients who were tested for the first time after seven days of life (i.e. beyond birth PCR), 5715 (59.6%) were tested between day eight and week 12 of life (median = 10 weeks; interquartile range [IQR] = 8–11 weeks). The proportions of male (49.9%) and female patients (49.8%) were similar, with the sex of 149 (0.3%) patients being unknown.

### Turn-around time

**FIGURE 2:**
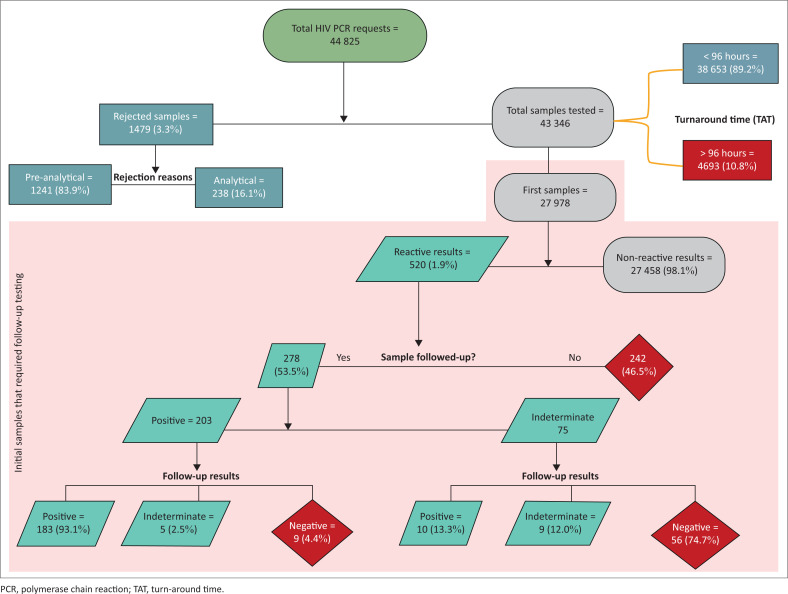
Numbers of HIV PCR requests, rejected samples, and follow-up tests conducted at Tygerberg Hospital, Cape Town, South Africa, between July 2017 and June 2019.

In total 38 653 (89.2%) test results were within the 96-h laboratory TAT cut-off (median = 44 h; IQR = 31–64), while the remaining 4693 (10.8%) were not (median = 116 h; IQR = 106–137) (Figure 2).

However, for the clinical TAT, only 33 245 (76.7%) samples were signed out within 96 h (median = 57 h; IQR = 47–73), while 10 101 (23.3%) test results were released after 96 h (median = 119 h; IQR = 105–140). The median TAT from sample collection to sample registration was 17 h (IQR = 8–24).

### Follow-up HIV PCR test results

Of the 520 (1.9%) reactive initial results, approximately half were followed up by testing of a subsequent sample. Similarly, 9409 (50.3%) of the initial negative results had subsequent samples tested, with only 1264 (13.4%) of these tested at 10 weeks of age (6–12 weeks of life) as per the national HIV testing guidelines. Nine (0.7%) of these 10-week samples had positive or indeterminate results. It should be noted that some of the follow-up results may have not been linked to the initial samples and may have been missed in this analysis. Of the 520 initial reactive tests, 251 (48.3%) were tested at birth, with 62.9% (*n* = 158) of those subjected to confirmatory testing. The remaining 269 (51.7%) were tested after seven days of life, and 44.6% (*n* = 120) of those had confirmatory testing conducted.

The reactive results were further analysed to assess the agreement between initial and confirmatory results. One hundred and eighty-nine (93.1%) patients with initial positive results were also positive following confirmatory testing, while five (2.5%) were indeterminate, and nine (4.4%) had discordant results (negative on follow-up testing) (Table 2). Further scrutiny of these nine results revealed that all of them had a third follow-up HIV PCR test done, with four testing positive and five testing negative. Of the 75 patients with initial indeterminate results who had a confirmatory sample tested, 56 (74.7%) had negative results. Of these, 30 (53.6%) had a third HIV PCR follow-up test done, 24 (42.9%) had no third follow-up HIV PCR or HIV viral load test conducted, while two patients had HIV viral load follow-up tests conducted, rather than an HIV PCR test (Table 3). Among the 30 patients who had a third HIV PCR test conducted, 29 (96.7%) were negative. One of two patients who had HIV viral load follow-up tests done had detectable HIV viral load (862 328 copies per millilitre). The presence of detectable HIV nucleic acid in this quantitative molecular test is confirmatory of HIV infection.

**TABLE 2:** Comparison of results of the initial reactive infant polymerase chain reaction tests and confirmatory tests conducted at Tygerberg Hospital, Cape Town, South Africa, between July 2017 and June 2019.

Initial test results that were followed up	Confirmatory test results						Total
	Positive		Indeterminate		Negative		
	*n*	%	*n*	%	*n*	%	
Positive	189	93.1	5	2.5	9	4.4	203
Indeterminate	10	13.3	9	12.0	56	74.7	75

**Total**	-	-	-	-	-	-	**278**

**TABLE 3:** Follow-up tests for samples that initially tested indeterminate at Tygerberg Hospital, Cape Town, South Africa, between July 2017 and June 2019.

Follow-up testing	Indeterminate + Negative (1st & 2nd HIV PCR)	Indeterminate + Indeterminate (1st & 2nd HIV PCR)
3rd HIV PCR done	30	6
Reactive 3rd HIV PCR	1	3
Non-reactive 3rd HIV PCR	29	3
No 3rd HIV PCR or HIV VL done	24	2
No 3rd HIV PCR, but HIV VL done	2	1
No 3rd HIV PCR, but detectable HIV VL	1	1
Both HIV PCR and HIV VL done	10	5
Non-reactive HIV PCR, but detectable HIV VL	1	0

**Total**	**56**	**9**

VL, viral load; PCR, polymerase chain reaction.

### Urgency of follow-up samples

Of the 278 follow-up tests conducted, 147 (52.9%) were performed more than seven days after the initial test (mean: 82 days; range: 8–606 days). Among the 131 (47.1%) follow-up samples tested within the first seven days of life, only 52 (39.7%) were tested within three days of the initial test.

## Discussion

In this study, we determined that 3.3% of infant HIV PCR requests were rejected, and that the majority of the test results met the TAT requirements. Infant HIV PCR requests were rejected for various reasons, mainly due to duplicated requests, insufficient sample volumes, and loss of specimen in transit. These rejections may occur when more than one clinician requests the same test for the same patient, when a sample is received in the laboratory with a volume lower than that required for the test, or when test requests are not accompanied by the patient’s sample.

The proportion of rejected samples is concerningly high and negatively impacts the early diagnosis and management of HIV-positive infants. Comparative data from other settings is limited, thus making it difficult to determine the rejection threshold across laboratories.

An overall appraisal of the TAT showed that the Tygerberg virology laboratory meets the standards set by the NHLS National Strategic Plan. However, the average laboratory TAT was longer when only positive and indeterminate results, which were the focus of our study, were considered; 14.9% of these results were released after the required 96 h. The clinical TAT was also longer, with only 76.7% of results released within 96 h. The clinical TAT may thus require some revisions to enable the expedition of early infant diagnosis and ART initiation. Point-of-care HIV PCR testing has been shown to accomplish same-day diagnosis for infants,^28^ with a significantly higher number of infants accessing confirmatory testing,^29^ thereby assisting with rapid ART initiation.

This study also found that only 39.7% of the reactive confirmatory tests were done within three days after the initial test. On average, most newborns would have been discharged from the hospital within three days of birth. If HIV birth PCR test results are received within this period, newborns with positive results are likely to be initiated on ART before being discharged, thus reducing the number of patients lost to follow-up.^28^

Although new mothers are required to visit the clinic within 6 days after birth, the 6-day maternal postnatal clinic visit was reported to be at 58.0% in the Western Cape between 2017 and 2019.^30^ The delay in follow-up testing (over seven days after initial testing) in 59.3% of patients in this study may be as a result of this low uptake or because our 96-h TAT only accounts for the processes within the laboratory.

While 1.9% of initial test results were reactive, only 53.5% of these had the prescribed confirmatory test done. It is unknown whether the other reactive results (46.5%) had confirmatory tests done, and this may mean that many HIV-positive patients may not have been initiated on ART, or that a substantial proportion would have been started on ART without confirmatory test results, effectively exposing some uninfected children to lifelong ART. The 53.5% follow-up test rate is similar to the 53.0% confirmatory test rate reported in a study in Tshwane, South Africa.^31^ This proportion may be different from what is seen in clinical practice^31^ as some of the patients, particularly those aged > 18 months, may have had either HIV viral load or serological follow-up tests. These patients would have been missed in our study, as we focused specifically on HIV PCR results.

The discordant negative results in 4.4% of patients with an initial reactive result indicate that these initial reactive results may have been false reactive or may be due to a rapid decline in HIV nucleic acid concentration following exposure to highly active ART in infants receiving antiretroviral drugs (as prevention of mother-to-child transmission or combination ART), making it difficult to make a definitive diagnosis of HIV infection.

Most children with indeterminate results tested negative on subsequent follow-up tests. Again, this could either indicate that a large proportion of these indeterminate results were initially false reactive, or may indicate rapid HIV nucleic acid decay in children who are receiving ART for prophylaxis, which may be two or three drugs in high-risk cases.^4,32^ It should, however, be noted that the indeterminate cut-offs used in this study were based on the reproducibility of a particular assay, with a particular chemistry and software, and within a different prevention of mother-to-child transmission context. These were later improved. Recently, HIV diagnostic criteria have been revised to address this diagnostic dilemma.^23,24,25,26^

Only 13.4% of patients with negative birth PCR results received a follow-up test within 10 weeks as per schedule; about half (50.3%) were later followed up after 10 weeks. This is consistent with other similar local studies on 10-week HIV PCR follow-up tests.^33^ At ten weeks, 0.7% of the infants in this study tested positive or indeterminate on HIV PCR, which is lower than the national HIV prevalence of 0.9% and the National Strategic Plan target of 1.3% at 10 weeks.^30^ It is, however, higher than the reported Western Cape rate of 0.5%.^30^ It should be noted that while these patients could have been missed at birth following intrauterine infection, they also could have been infected either perinatally or postnatally.

Just over half of the infants who required confirmatory PCR testing received such tests. It remains unclear whether the patients who were not followed up were HIV positive or not. A small proportion of babies received the 10-week follow-up test. The patients who did not receive this test may only be seen in the hospital later in life when they are sick. Clinician education on HIV testing guidelines may help to reduce this number, and strengthening systems to reduce the time between first and confirmatory HIV PCR tests could also be beneficial.

Most of the indeterminate results were negative on the second and third follow-up tests. It was not known if patients were receiving prevention of mother-to-child transmission regimens or combination ART during the periods of observation, as that may have resulted in undetectable HIV nucleic acid. The new early infant diagnosis criteria, as well as a separate and independent HIV PCR testing platform, may help resolve the challenges presented by indeterminate HIV PCR results.

### Limitations

Our interpretation of the TAT data did not account for the current workflow practice, in which HIV PCR testing is processed only during weekdays (Monday to Friday). The fact that no tests are done on weekends may result in TAT variability. This study is thus not indicative of laboratories that operate a shift system (i.e., a 24-h service). Occasionally, HIV PCR results also fail to transmit to the LIS, further delaying the TAT.

Another limitation of the study was the classification of indeterminate results using outdated criteria that have since been revised. Some of these results may now be re-classified as positive.

Follow-up tests were reconciled using a unique laboratory code (medical record number) for each patient. However, we noted that some patients may have erroneously had more than one medical record number. This could have occurred in instances where samples from babies were labelled with the mother’s name (i.e., baby of XYZ) and then later labelled with the baby’s name, resulting in non-reconciled patient profiles. This might have contributed to the under-reporting of follow-up tests, as these would have been missed. We, however, compared data provided on actual request forms with those captured by the LIS and found them to match.

Also, some patients could have moved to other health facilities outside of our testing site. It must be noted that this study was limited to the Tygerberg Hospital Virology Laboratory, and thus may not be representative of the whole Western Cape region (i.e. Groote Schuur Hospital and Green Point Complex). The magnitude of this data concern is unknown due to our inability to assess the number of follow-up results that were not linked with the initial patient results.

### Conclusion

A high proportion of infant HIV PCR requests were rejected for various reasons. HIV PCR testing TAT at the Tygerberg laboratory is at par with the national requirements. However, this 96-h laboratory TAT may need to be revised as it may negatively impact the number of children who receive confirmatory testing after a positive result. A larger study is recommended for a clearer appreciation of the HIV PCR testing challenges.
